# Real-time noise cancellation with deep learning

**DOI:** 10.1371/journal.pone.0277974

**Published:** 2022-11-21

**Authors:** Bernd Porr, Sama Daryanavard, Lucía Muñoz Bohollo, Henry Cowan, Ravinder Dahiya

**Affiliations:** 1 Biomedical Engineering, James Watt School of Engineering, University of Glasgow, Glasgow, United Kingdom; 2 Bendable Electronics and Sensing Technologies (BEST) Group, James Watt School of Engineering, University of Glasgow, Glasgow, United Kingdom; University of Southern Denmark, DENMARK

## Abstract

Biological measurements are often contaminated with large amounts of non-stationary noise which require effective noise reduction techniques. We present a new real-time deep learning algorithm which produces adaptively a signal opposing the noise so that destructive interference occurs. As a proof of concept, we demonstrate the algorithm’s performance by reducing electromyogram noise in electroencephalograms with the usage of a custom, flexible, 3D-printed, compound electrode. With this setup, an average of 4dB and a maximum of 10dB improvement of the signal-to-noise ratio of the EEG was achieved by removing wide band muscle noise. This concept has the potential to not only adaptively improve the signal-to-noise ratio of EEG but can be applied to a wide range of biological, industrial and consumer applications such as industrial sensing or noise cancelling headphones.

## Introduction

Low signal-to-noise ratios (SNR) exist in many application domains, such as communications, acoustics or biomedical engineering. In particular, the Electroencephalogram (EEG) [1–3] has a low SNR ratio because of its low amplitudes, in the range of a few *μV*, which are contaminated by numerous sources, often orders of magnitude larger than the EEG signal itself [4]. In this work, we target EEG as an example application and remove non-stationary electromyogram (EMG) noise. However, this concept of algorithmic SNR enhancement is not limited to this particular use case.

There are two categorical approaches to increasing the SNR of an EEG signal: real-time processing and offline post-processing. Concerning the latter, by far the most popular approach is principal component analysis (PCA) or independent component analysis (ICA) [5–9]. PCA and ICA methods pre-analyse the raw signals in order to identify and separate the signal and noise components. This analysis is offline, requires the signal and noise relationships to be constant over time, and demands high computational power.

Real-time algorithms, on the other hand, filter the EEG signals as they arrive, sample by sample, and do not rely on offline pre-analysis, for example, bandpass filters, the short time Fourier Transform or wavelet transform [10–12]. These techniques still require prior knowledge of the noise to tune the filter parameters. However, muscle noise is non-stationary due to both voluntary and involuntary contractions of surrounding facial muscles. A solution to this problem is real-time adaptive filtering in which the noise is removed by an adaptive algorithm [13–15]. In cases where EEG electrodes are placed on top of the head (i.e. around *C*_*z*_), one can assume that noise polluting the EEG originates from further afield and affects all electrodes in equal measure, while the EEG signals originate locally [16]. A second auxiliary electrode can be used for measuring the noise solely, this can then be *subtracted* from the main EEG electrode signal. The most popular design for such an auxiliary electrode is a ring-shaped electrode around the main EEG electrode where the noise is simply subtracted, this is called the “Laplace operator” [16–20]. While the idea of simply subtracting the noise is perfect in theory, in practice, the relationship between the EEG generated in the brain and the resulting signals at the electrodes are complex and dynamic. This calls for a smart, compound electrode that implements an adaptive filter to continuously learn about the changing signal and noise conditions.

In this paper, we present a proof of concept for a novel, compound electrode which is inexpensive and readily manufacturable, in combination with a new deep learning algorithm. This system adaptively removes the noise from the EEG by algorithmically creating an opposing signal to the noise which is, in turn, used to cancel out the noise. This is demonstrated below by the removal of wideband muscle (EMG) noise.

## Methods

### General signal requirements

Let us consider a signal $\tilde{d}\left[n\right]$ measured with an ordinary electrode placed on the head of a subject:

$$\begin{array}{c} \tilde{d}\left[n\right]=\underset{r[n]}{\underset{︸}{b[n]+m[n]}}+c\left[n\right] \end{array}$$ (1)

which is a superimposition of three signals:

1. *c*[*n*] is the signal of interest generated by a stimulus or voluntarily, for example, in the setting of a brain-computer interface (BCI),
2. *b*[*n*] is the background EEG activity which is involuntary and unaffected by the stimulus, and
3. *m*[*n*] is the accumulation of all artefacts, in particular muscle activity (EMG). The latter two signals form the total baseline noise *r*[*n*] contaminating the EEG component *c*[*n*] which is of interest for diagnostics or BCI applications.

The task is now to reduce *r*[*n*] as much as possible with the help of an opposing signal which ideally eliminates the noise from $\tilde{d}\left[n\right]$. As outlined in the introduction, we assume that the EEG originates *locally* from a small surface area of the head and that artefacts originate further afield and, therefore, they have a *global* and uniform strength across the scalp of the subject.

**Fig 1:**
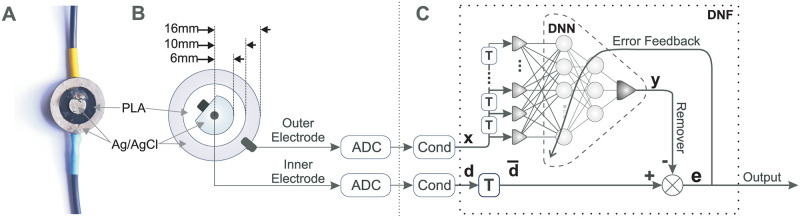
Electrode and deep neural filter. A: Photo of the manufactured compound electrode. The top wire (yellow) connects to the inner electrode and the bottom wire (blue) to the outer ring electrode. B: Top schematic view of the new compound electrode showing the inner electrode and the outer ring electrode. C: Signal processing of the two signals originating from the inner and outer ring electrodes: ADC = Analogue Digital Converter, Cond = standard signal conditioning such as high-pass filtering and 50 Hz removal. T = time delay.

The use of a second and linearly independent measurement would provide more information about the relationship between the global noise and the local EEG signal. Consequently, a compound electrode (Fig 1A and 1B) is designed with the addition of an annular ring-like outer electrode around the inner electrode, which acts as the noise reference. Thus, the compound electrode collects two separate signals:

$$\begin{array}{ccc} \tilde{d}\left[n\right]= & r[n]+c[n]\; & \text{Inner}\;\text{electrode}\;:\;\text{signal}+\text{noise} \end{array}$$ (2)

$$\begin{array}{ccc} \tilde{x}\left[n\right]= & h\left[n\right]*(r[n]+\alpha \cdot c[n]) & \text{Outer}\;\text{Ring}\;\text{electrode}\;:\;\text{noise}\;\text{reference} \end{array}$$ (3)

where 0 < *α* ≪ 1 models the crosstalk between the inner $\tilde{d}\left[n\right]$ and outer $\tilde{x}\left[n\right]$ electrode signals, as the signal *c*[*n*] of the inner electrode *d*[*n*] will also stray into the outer ring. The noise in turn should ideally be present at both the inner part of the electrode $\tilde{d}\left[n\right]$ and the outer, ring electrode $\tilde{x}\left[n\right]$ but in practice, it will be a filtered version and is modelled with the filter *h*[*n*]. The goal of the learning algorithm is to render the signal from the inner electrode $\tilde{d}\left[n\right]$ as noise-free as possible so that ideally only *c*[*n*] remains. Naively, one could simply subtract the outer electrode signal $\tilde{x}\left[n\right]$ from the inner one $\tilde{d}\left[n\right]$ to obtain a noise-free EEG but in practice, this is not possible because of changing noise-characteristics which are modelled here with the filter *h*[*n*]. Instead, we present a new machine learning algorithm which learns in real-time (i.e., when the data is being collected) to alter the signal from the outer noise reference electrode $\tilde{x}\left[n\right]$ in such a way that it eliminates the noise from the inner electrode which then results in a noise-free EEG signal. In the next two sections, we describe the electrode and the deep neural filter algorithm, respectively.

### Fabrication of the compound electrode

To record both the noisy EEG and a noise reference, a new compound electrode was designed (Fig 1A and 1B). The physical design of the electrode was driven by durability, ease of manufacture and reliability. Polylactate acid (PLA) was chosen as the electrode material due to its compatibility, flexibility, and adhesive nature to silver/silver-chloride (Ag/AgCl) ink [21]. Ag/AgCl paste was selected for the conductive portion. Ag/AgCl was selected over alternative materials such as gold or stainless steel as it is conformable (in ink form) allowing easy application to the PLA and inexpensive. In addition, Ag/AgCl has a low half-cell voltage [22], meaning any oxidisation of the electrode will have a minimal effect on the sensitivity of the electrode. The combination of a flexible backing with conductive paste versus conventional, rigid, and often uncomfortable gold/platinum electrodes [23, 24], is advantageous as it allows for optimal skin-electrode contact. This optimal contact ensures minimal inter-electrode impedance resulting in an increased SNR for that electrode whilst also providing more comfort to the patient [25], making long-term monitoring applications more viable. The compound electrode consists of two raised ring portions separated by a channel. The PLA geometry was 3D printed and the surface areas of the different electrode compounds were:

$$\begin{array}{c} \begin{array}{cc} A_{\text{inner}\;\text{Ring}} & =\pi \cdot {\left(6\;\text{mm}\right)}^{2}=113\;{\text{mm}}^{2}\\ A_{\text{Outside}} & =\pi \cdot {\left(16\;\text{mm}\right)}^{2}=804\;{\text{mm}}^{2}\\ A_{\text{Inside}} & =\pi \cdot {\left(10\;\text{mm}\right)}^{2}=314\;{\text{mm}}^{2}\\ A_{\text{Outer}\;\text{Ring}} & =A_{\text{Outside}}-A_{\text{Inside}}\\ A_{\text{Outer}\;\text{Ring}} & \approx 490\;{\text{mm}}^{2} \end{array} \end{array}$$ (3)

When selecting the optimal surface areas, there is always a trade-off between localisation and signal strength. Increasing the area results in more contact area and thus receiving a stronger signal [26], but decreases the spatial resolution of the signal. In our application, we assume that EEG has a narrow spatial localisation and therefore requires a small surface area. We also assume that the noise has a broad spatial localisation as it is predominantly EMG artefacts perturbing the scalp all-across. An increase in the outer electrode area would, in theory, allow us to capture more EMG-noise for the algorithm to self-tune, however, as the signal strength is already orders of magnitude lower than the noise any realistic adaptation of surface area (given the necessity of comfort and localisation) would most likely result in negligible SNR enhancements.

A layer Ag/AgCl paste was deposited on each of the raised rings using a plastic spatula. The Ag/AgCl was then cured at 70°C for 1 hour. Fig 1A shows the final printed electrode with Ag/AgCl applied. Two wires were connected to each electrode substrate by melting the copper onto the flexible PLA geometry using a soldering iron. Next, pure silver paste and epoxy were applied to the contact point to ensure reliable electrical contact and solidify the connection, respectively. This electrode has proven to be robust and easy to both manufacture and integrate into a headband or EEG cap as a wearable device.

### Experimental setup for EEG recording

Ethical approval for this experiment was obtained from the ethics committee at the Institute of Neuroscience and Psychology, School of Psychology at the University of Glasgow, reference number 300210055. In total, 20 subjects were recruited. Subjects were instructed to read an information sheet detailing the experiments and were permitted to participate after providing written consent. Every participant signed two copies of the consent form, one for the investigator and another for the participant to keep. The ethical approval letter, the information sheet and the consent forms are bundled together with the open-access dataset [27]. The data was acquired using a two-channel data acquisition device (“Attys”, www.attys.tech) with the data acquisition programs attys-ep and attys-scope. Referring to the international 10–20 system, our compound electrode (see Fig 1A and 1B) was placed on the subject’s head at *C*_*z*_, with its *inner* part connected to the positive input of Channel 1 and its *outer* ring electrode to the positive input of Channel 2 of the Attys. The A2 electrode (standard adhesive electrode behind right ear) was connected to the negative input of Channel 1, and the A1 electrode (standard adhesive electrode behind left ear) was connected to the negative input of Channel 2 which also acted as ground. In the remainder of the paper we will just refer to the “inner” part and “outer” ring of the compound electrode and their corresponding signals (see Eqs 2 & 3). Each subject held two sessions with no intervals to guarantee consistent electrode signals:

1. In this session the subject generates *EEG polluted with EMG noise*. To achieve this the subject was asked to contract their jaw muscle every 15 secs for two minutes to generate EMG noise. The sampling rate was *f*_*s*_ = 500 Hz and was chosen to obtain a flat response in the EEG frequency band of 0…100 Hz due to the sigma-delta converter’s smooth roll-off towards the Nyquist frequency of 250 Hz.
2. This session is used to obtain the *signal power of a noise-free EEG*. Since the subject cannot be paralysed to obtain EMG-free EEG signals, evoked potentials have been chosen to average out EMG noise. P300 visually induced oddball stimuli were used to determine the pure EEG signal *c*[*n*] and its power (5 minutes). A black and white chequerboard inversion was presented every second and brightly-coloured horizontal bars as oddballs were randomly interspersed every 7 sec to 13 sec. The subject had the task of silently counting the number of oddball stimuli. The sampling rate was *f*_*s*_ = 250 Hz; since the evoked potentials have low-frequency components, only their peak power is of interest in this work.

We are now going to describe our new adaptive noise reduction algorithm which was then used to remove the EMG noise from the recordings of the different subjects.

### Deep Neural Filter (DNF)

Fig 1C shows the block diagram of our Deep Neural Network (DNN) which in conjunction with the additional building blocks becomes our novel Deep Neural *Filter* (DNF) to remove noise (see [28] for the source code). Recall that the deep network exploits the assumption that the signal from the outer electrode *x*[*n*] ideally just contains the noise and that the DNN learns to subtract it from the signal *d*[*n*] originating from the inner electrode at the summation node “X”.

The error signal *e*[*n*] of the network is also the final output of the DNF as is the case with LMS noise cancellation frameworks. This might appear counter-intuitive, as in classical applications of neural networks, the error *e*[*n*] is expected to converge to zero. At the same time, for filtering applications, the output is expected to be the clean signal. The key to resolving what appears to be a contradiction is to realise that before learning the output of the DNF *e*[*n*] is a superposition of both EMG-noise and the pure EEG-signal. The noise component is expected to converge to zero through learning, leaving only the clean EEG-signal available at the output. This is possible because the learning that takes place within the DNF network is not solely driven by the error feedback *e*[*n*], rather, it is driven by the correlation of the error feedback *e*[*n*] and the noise reference *x*[*n*]. If these two signals correlate, meaning some components of the noise is present at the output of the DNF, these shared components will be removed by the remover *y*[*n*]. This process will continue until the error feedback *e*[*n*] and the noise reference *x*[*n*] no longer correlate, meaning no components of the EMG-noise have remained at the output of DNF. This marks successful learning. In other words, the noise component of the output *e*[*n*] has converged to zero despite it being a non-zero signal, this remaining component is the clean signal. In practice, the noise reference *x*[*n*] often contains a certain amount of the pure EEG signal *c*[*n*] which results in a reduction of the EEG signal at the DNF output. On the other hand, any uncorrelated noise between inner and outer electrodes such as thermal noise (approx. $65\;\text{nV}=\sqrt{1.38064910^{-23}\;J/K\cdot 4\cdot 310\;K\cdot 1\;K\Omega \cdot 250\;\text{Hz}}$) or ADC-converter noise (approx. 100 nV) has no impact on learning and passes through the DNF.

As outlined above the goal is to reduce EMG noise. However, eye-blink artefacts and slowly changing electrode drift have much higher noise power than EMG. Given that we are interested in EMG, we need to provide the reference noise input *x*[*n*] with the muscle noise spectrum and remove the much more powerful low-frequency artefacts such as eye movement or baseline wander. We do this by employing a high-pass filter which captures the typical EMG spectrum which is flat above 20 Hz but slowly decays in power below 20 Hz [29] (see also Fig. AC in S1 Appendix). To force the DNF to learn the noise features of the EMG and not those of the EOG we set the 2nd order Butterworth high-pass for the noise reference *x*[*n*] to $f_{c_{x}}=5\;\text{Hz}$ which gives a shallow rise in the passband. The high-pass filter frequency for the inner signal *d*[*n*] is not critical and was set to $f_{c_{d}}=0.5\;\text{Hz}$ to simply remove the DC from the DC-coupled ADC converter so that all signals are DC-free:

$$\begin{array}{ccc} d[n] & = & \gamma \cdot {\text{HP}}_{f_{c_{d}}}\left[n\right]*\text{BS}\left[n\right]*{\text{LP}}_{\text{ADC}}*\tilde{d}\left[n\right] \end{array}$$ (5)

$$\begin{array}{ccc} x[n] & = & \gamma \cdot {\text{HP}}_{f_{c_{x}}}\left[n\right]*\text{BS}\left[n\right]*{\text{LP}}_{\text{ADC}}*\tilde{x}\left[n\right] \end{array}$$ (6)

where ${\text{HP}}_{f_{c_{d}}}\left[n\right]$ and ${\text{HP}}_{f_{c_{x}}}\left[n\right]$ are the 2^nd^ order high-pass Butterworth filters for the inner and outer electrodes, respectively. BS[*n*] is a 2^*nd*^ order Butterworth notch filter against powerline interference at 50 Hz. LP_ADC_ is the low-pass characteristic of the sigma-delta converter with a cutoff at about half the sampling rate. The gain was set to *γ* = 1000 so that each neuron in the input layer of the neural network received values of approximately ±0.2V. The DNF uses as activation function tanh which saturates for values above approximately one but the input range of *x*[*n*] at ±0.2 will steer clear of any hard saturation. This also prevents vanishing gradients as the derivative of tanh will be close to one in this regime and will be far off from becoming zero which only happens when tanh saturates. On the other hand at 0.2 the tanh is in its non-linear regime and the network will use its non-linear properties.

Inspired by a Finite Impulse Response (FIR) filter, we send the signal of the outer electrode *x*[*n*] through a tapped delay line with

$$\begin{array}{c} N_{{\text{taps}}_{x}}=\frac{f_{s}}{f_{c_{x}}} \end{array}$$ (7)

taps and then feed it into the Deep Neural Network (see Fig 1C). The signal *d*[*n*] is delayed by $N_{{\text{taps}}_{x}}/2$ so that the DNN has time to react to pulse-like muscle artefacts arriving at *x*[*n*].

The output of the Deep Neural Network *y*[*n*] is then used to remove the noise from *d*[*n*]:

$$\begin{array}{c} e[n]=d[n]-y[n] \end{array}$$ (8)

Ideally, this is the noise-free EEG which is at the same time the error signal for the DNN and back-propagated in real-time. Learning is “on” (i.e. in effect) at *all times*, meaning, the network adjusts to the changes in the electrode contact as they happen.

The network used for DNF is a feed-forward neural network with fully connected layers designed with *L* = 6 layers. The number of neurons *I*(ℓ) per layer index ℓ is calculated as:

$$\begin{array}{ccc} b & = & e^{\frac{\text{ln}N_{{\text{taps}}_{x}}}{L-1}} \end{array}$$ (9)

$$\begin{array}{ccc} I(\ell ) & = & \lfloor \frac{N_{{\text{taps}}_{x}}}{b^{\ell -1}}\rfloor \;\text{where}:\;\ell :1,\ldots ,L \end{array}$$ (10)

which guarantees that the output layer consists of exactly one neuron which then generates the “remover” *y*[*n*]. In our case with $N_{{\text{taps}}_{x}}=50$ inputs to the DNF this results in: *I* = 50, 22, 10, 4, 2, 1 neuron(s) per layer which means that the first layer is fully connected with the same number of neurons to the delay line and then the number of neurons are reduced in the form of a funnel as done in auto-encoders.

The weights of the neurons were initialised to a random value in the range of (0, 1]. Eq 11 below shows the forward propagation of the outer electrode signal *x*[*n*] through the first layer of the network:

$$\begin{array}{c} a_{j}^{0}\left[n\right]=\text{tanh}\left(z_{j}^{0}\left[n\right]\right)=\text{tanh}(\sum_{k=0}^{N_{{\text{taps}}_{x}}}\left(\omega_{kj}^{0}x\left[n-k\right]\right)) \end{array}$$ (11)

where *x*[*n*−*k*] is the filtered signal from the *k*^*th*^ tap of the delay line for the outer electrode signal (Fig 1). In contrast to deep networks performing classification we filter a DC-free signal. For that reason, there are no bias weights to keep the processing DC-free. The activation function is tanh because it is ideal for signal processing: it is linear at the origin and becomes non-linear with growing signal strength so that learning can self-tune the non-linear processing. In the frequency domain, this means the network self-tunes the number of harmonics it is adding to the signals and thus to the remover *y*[*n*].

Similarly, these activations propagate through the deeper layers in the network:

$$\begin{array}{c} a_{j}^{\ell }\left[n\right]=\text{tanh}\left(z_{j}^{\ell }\left[n\right]\right)=\text{tanh}(\sum_{i=0}^{I(\ell )}\omega_{ij}^{\ell }a_{i}^{\ell -1}\left[n\right])\;\text{where}:\;\ell :1,\ldots ,L-1 \end{array}$$ (12)

Finally, in the output layer, this weighted sum results in the generation of the “Remover” signal *y*[*n*]:

$$\begin{array}{c} y\left[n\right]=\text{tanh}\left(z_{0}^{L}\left[n\right]\right)=\text{tanh}(\underset{z_{0}^{L}\left[n\right]}{\underset{︸}{\sum_{i=0}^{I(L)}\omega_{i}^{L}a_{i}^{L-1}\left[n\right]}}) \end{array}$$ (13)

The “Remover” signal *y*[*n*] then ideally cancels out the noise from the inner electrode *d*:

$$\begin{array}{c} e[n]=d[n]-y[n] \end{array}$$ (14)

As explained in previous sections, the output of the DNF *e*[*n*] is the noise-free EEG signal and is also used for the learning of the neural network which is done by error backpropagation:

$$\begin{array}{c} \delta^{L}=e\left[n\right] \end{array}$$ (15)

where *δ*^*L*^ is the error in the output neuron which is then backpropagated. For deeper layers this is defined through the back-propagation as:

$$\begin{array}{c} \delta_{j}^{\ell }=\sum_{k=1}^{K}\left(w_{jk}^{\ell +1}\delta_{k}^{\ell +1}\right)\cdot {\text{tanh}}^{\prime }\left(z_{j}^{\ell }\right)\;\text{where}:\;\ell :L-1,\ldots ,0 \end{array}$$ (16)

Remember that we keep the weighted sum $z_{j}^{\ell }$ well below one so that the derivative ${\text{tanh}}^{\prime }\left(z_{j}^{\ell }\right)$ stays close to one preventing vanishing gradients.

The changes in weights that cause the optimum reduction in noise are dictated by gradient descent rule:

$$\begin{array}{c} \Delta \omega_{ij}^{\ell }=\eta a_{i}^{\ell -1}\cdot \delta_{j}^{\ell } \end{array}$$ (17)

where *η* is the learning rate.

It’s important to note that the effective learning rate *η*_e_ directly scales with the amplitude of the noise reference *x*[*n*]:

$$\begin{array}{c} \eta a_{i}^{\ell -1}\cdot \delta_{j}^{\ell }\equiv \underset{\eta_{e}}{\underset{︸}{\eta x[n]}}\cdot e\left[n\right] \end{array}$$ (18)

To have a constant effective learning rate one could either normalise the noise reference *x*[*n*] or adjust the learning rate dynamically if the average amplitude of *x*[*n*] is changing. In this work, we directly set the learning rates to accommodate the two different noise reference amplitudes of *x*[*n*] for the P300 task (*η* = 10) and the jaw muscle task (*η* = 2.5) so that the effective learning rates were the same between the two tasks. The above equation also shows that learning converges when the correlation between the noise reference *x*[*n*] and the error signal *e*[*n*] weakens, meaning no frequency components of the noise present in the outer electrode signal remain in the output of the DNF filter and thus the noise has been removed.

### Calculating the signal-to-noise ratio

The *signal* from the inner electrode (Eq 2) is a mix of baseline EEG, EMG and the consciously created EEG signal *c*[*n*]. To have a realistic estimate of *c*[*n*] we use the power of the primary peak of the P300 evoked potential. To reduce the noise of the peak we took the median power between 300 ms and 500 ms which takes into account the 100 ms latency of the wireless transmission between the ADC and the P300 software. This means that in terms of the power of the signal, we can think of the P300 as a pulse at *t* = 300*ms* which could be detected too, for example, by setting up a P300 speller. Note that the median over this time interval will underestimate the power slightly. However, this is deliberate because real-time BCI systems hardly average over 5 minutes (they do so over seconds), meaning they deal with much lower signal strengths for *c*[*n*] and thus using the median filter corrects for overly optimistic signal strength.

In terms of *noise*, we are interested in the power of the EMG generated by facial muscles and the jaw muscle but not in the low-frequency band such as electrooculogram (EOG) or electrode drift. To assess mainly EMG and underlying EEG background noise we calculated the periodogram with the Welch method which had a window length at the sampling rate giving the power density in bins of 1 Hz. The power density samples from 5 Hz…125 Hz were summed up given the total noise power in the frequency band between 5 Hz and 125 Hz.

The SNR is then calculated as:

$$\begin{array}{c} \text{SNR}=\frac{\text{median}(v_{P300,\pm 100\;\text{ms}}^{2})}{\sum_{k=5\;\text{Hz}}^{125\;\text{Hz}}\text{Welch}\left(v\right)\left[k\right]} \end{array}$$ (19)

where *v* can be one of the following signals: a) the inner electrode signal *d*[*n*], b) the output *e*[*n*] of the DNF, c) the output of a standard LMS-based FIR filter, and d) the output of the Laplace operator by directly subtracting the raw outer electrode signal $\tilde{d}\left[n\right]$ from the inner $\tilde{x}\left[n\right]$ one.

The recordings from the 20 subjects were then checked for valid EEG/EMG-signals and if deemed acceptable, processed one by one by the deep neural filter where the network had to learn from scratch (random re-initialisation of weights) for every subject. All parameters stayed the same for all subjects.

## Results

**Fig 2:**
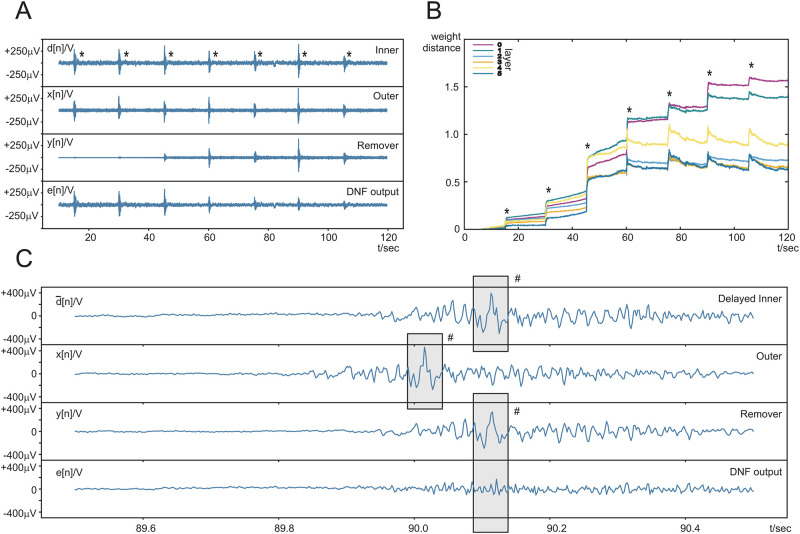
Signals and weight development from subject 10. The subject was asked to contract their jaw muscles every 15 seconds. The jaw contractions are indicated with a “*”. A: Four signal traces, namely: the inner electrode signal *d*[*n*] which carries a mix of EEG and EMG, the outer electrode signal *x*[*n*] which is the noise reference, the output of the DNN or the “remover” *y*[*n*], and the output of the DNF *e*[*n*] which is both the output and the error signal. B: Weight development: shows the Euclidean weight distance from the initial weights of the 6 different layers over time. C: Detailed plot of the same signals as panel A between 89.5 s and 90.5 s. The jaw clench starts at about 89.8 s.

The data from the 20 subjects [27] were examined for electrode failure or strong external interference. Subject 2 had a faulty *x*[*n*] channel and subject 5 had unexplained strong artefacts possibly from a power surge. Thus, the results of subjects 2 and 5 were excluded but the data from all other subjects are presented and analysed in this section. Before presenting the results of all subjects, as an instructional example, we focus on subject 10 to gain a deeper understanding of learning behaviour. Fig 2A shows the progress of real-time learning of the DNF over a period of 2 mins for subject 10. “Inner” shows the signal *d*[*n*] of the inner part of the compound electrode. The voluntary jaw muscle contractions every 15 seconds are clearly visible and indicated with a “*”. Between the muscle contractions, the signal is most likely a mix of baseline EEG and lower amplitude involuntary facial muscle (EMG) activity. The “Outer” trace shows the signal from the outer ring electrode *x*[*n*] where the EMG bursts, caused by the jaw muscles, are clearly visible. These two signals, “Inner” and “Outer”, are then sent into the Deep Neural Filter (DNF). The most important internal signal is the “Remover” *y*[*n*] which eliminates the noise (Eq 14). The result of the subtraction *e*[*n*] can be observed in the bottom trace “DNF output”.

Processing of the two minutes of EEG recording at 500 Hz took 105 s on an Intel(R) Core(TM) i7–5600U CPU running at 2.60 GHz and shows that the DNF filter is real-time on a general purpose processor without the need for special GPU hardware. The DNN has in total 6 layers and their weight development, related to Fig 2A is shown in Fig 2B over the two minutes. Plotted is the weight distance from the initial randomly initialised weight values. Learning is fastest during the jaw muscle contractions as the noise reference *x*[*n*] has a higher amplitude and thus the effective learning rate is higher (Eq 18) during the jaw muscle bursts but also continues to learn between EMG bursts at a lower rate. From about 60 seconds, learning has stabilised with only smaller adjustments to the weights till the end of the experiment. Because the filter acts in a closed loop corrective action happens where the weights shrink again after a jaw contraction indicating that jaw muscle recruitment and involuntary muscle activity cause slightly different correlations so that the network re-adjusts.

Fig 2C shows a zoomed-in segment of Fig 2A between 89.5 s and 90.5 s at the onset of a jaw clench at about 89.8 s. To see how the removal process works the first trace $\bar{d}\left[n\right]$ shows the delayed version of the inner electrode signal (see Fig 1). The noise reference *x*[*n*] from the outer electrode is shown as it is fed into the DNF and then enters its tapped delay line. The DNF then creates the remover signal *y*[*n*] which then cancels out noise in $\bar{d}\left[n\right]$ which is diminished at the output of the DNF *e*[*n*] in the bottom trace which is also the error signal for training. As a detailed example of how the removal process works the section marked with the “#” has been chosen. Remember that the DNF removes anything which is present in both the contaminated signal *d*[*n*] and the noise reference *x*[*n*]. This can clearly be seen that the large peak is present in both the contaminated signal and the noise reference. Thus, the DNF learns to remove this peak and leaves the rest of the signal intact. Note that *e*[*n*] is also the error signal which is no longer correlated with the noise reference *x*[*n*] which averages out in the learning rule Eq 17 and consequently, the weights stabilise which is the case at about 90 s into learning (Fig 2B).

**Fig 3:**
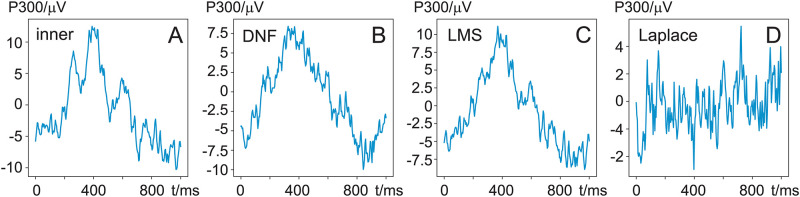
P300 averages from subject 10. While looking at a chequerboard that inverted every second, the subject was presented with oddball stimuli every 7sec-13sec with a random pattern. The recording was 5 minutes long. A: the event-triggered average from the inner electrode *d*[*n*], B: the event-triggered average from the output *e*[*n*] of the DNF, C: the output from the LMS filter (adaptive FIR filter) and D: from the Laplace filter: $\tilde{d}\left[n\right]-\tilde{x}\left[n\right]$ with DC and 50 Hz removed after the subtraction operation.

To calculate the SNR, the *power of the signal* and *the power of the noise* (see Eq 19) have to be calculated separately. First, we focus on the *signal power*. As outlined above, the signal power is estimated by calculating the power of the primary P300 peak, measured during experimental session 2. Note that there is no need to send the EEG containing the P300 through the DNF as the event-related averaging eliminates the EMG noise. However, as a sanity check we inspected the P300 peaks before and after noise reduction, this is shown in Fig 3 for subject 10. P300 has a low frequency by nature and with the DNF and LMS removing the higher EMG frequencies, one expects that the shape of the P300 is not substantially altered which is confirmed by comparing the unfiltered Fig 3A and filtered P300 Fig 3B and 3C. The original EEG, the DNF output and the LMS filter all yielding clearly identifiable peaks and their squared values represent the signal power. Comparing the P300 from the original electrode signal (A) with that of the DNF (B) output the P300 peak of the DNF is reduced by approximately 1/4 (from 10 *μ*V to 7.5 *μ*V) while the LMS filter causes virtually no reduction. This means that the DNF filter needs to reduce the noise even more than the LMS to achieve an overall SNR improvement, as the DNF diminishes the P300 peak. However, this is expected as there is certainly crosstalk between the inner electrode and the outer ring electrode (Eq 3) where EEG from the inner electrode is also partially present at the outer electrode. Since the DNF removes anything which is present in both the noise reference *x*[*n*] and its input signal *d*[*n*] it will treat the *α* > 0 crosstalk of the EEG signal at the outer electrode as noise and consequently reduces the amplitude of the noise-free EEG at its output. Finally, it is evident that the Laplace operator completely removes the P300 peak effectively rendering the SNR calculations for a pure Laplace operator impossible. Having calculated the *signal* power for the SNR, we move on to consider the *noise* power.

**Fig 4:**
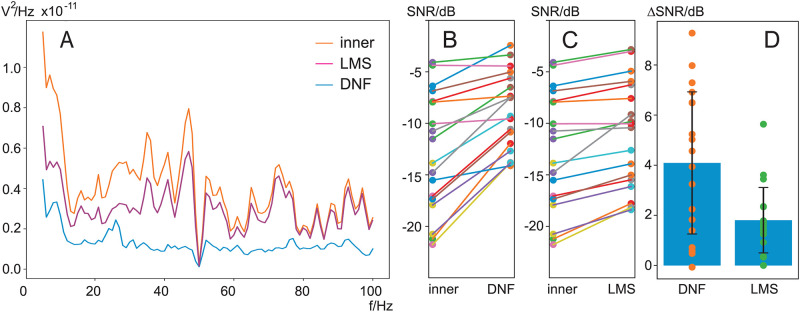
Noise density and SNR calculations. A: Noise power density in bins of 1 Hz at the inner electrode *d*[*n*], the output of the DNF *e*[*n*], and the output of the standard LMS-based adaptive FIR filter. B: SNR in dB calculated with Eq 19 at the inner electrode *d*[*n*] and the output *e*[*n*] of the DNF for every subject. C: SNR in dB calculated with Eq 19 for the standard LMS-based adaptive FIR filter for every subject. D: The SNR differences from C) and D) for DNF (ΔSNR_DNF_ = 4.1±2.8 dB) and LMS-based FIR filter (ΔSNR_LMS_ = 1.8±1.3 dB).

In this section we calculate the *noise power* according to Eq 19. Fig 4A shows the power spectral density of the signal from the inner electrode *d*[*n*] and the output from both the DNF and a standard LMS-based adaptive FIR filter. The DNF filter achieves a nearly flat reduction of the noise to about 0.1⋅10^−11^ *V*^2^/Hz for frequencies above 10 Hz while the original noise from the inner electrode *d*[*n*] fluctuates widely between 0.2 ⋅ 10^−11^…0.8 ⋅ 10^−11^ *V*^2^/Hz. The FIR filter tuned by LMS, being a linear filter with just one layer, also achieves a noise reduction but falls short by simply reducing the spectral components in a nearly proportional way and is not able to eliminate the noise peaks, for example at 35 Hz, 40 Hz or 45 Hz, but only reducing them. Given that both the DNF and the FIR filter tuned by LMS receive the same input signals the smooth frequency spectrum of the DNF output is clearly a distinctive feature of this filter.

The individual SNR changes between the different subjects are shown in panel B and C for DNF and LMS filters, respectively. It is evident that the worst SNR at −20*dB* can be improved most where strong EMG bursts from the jaw muscles are eliminated as shown in Fig 2. For some subjects the improvement has been marginal and this might be due to poor electrode contact and thus little correlation between the inner and outer electrodes.

To test if the noise reduction has been statistically significant, we calculated the SNR for every subject before and after filtering (in dB) to obtain the SNR improvement:

$$\begin{array}{c} \Delta \text{SNR}={\text{SNR}}_{\text{inner}}-{\text{SNR}}_{\text{DNF}}/\text{LMS} \end{array}$$ (20)

Fig 4D shows the SNR improvements for both the DNF and the LMS filter. Both our new DNF (*p* = 0.000013) and a LMS-tuned adaptive FIR filter (*p* = 0.000192) significantly improved the SNR but the DNF is significantly better than the LMS filter (*p* = 0.000026).

## Discussion

The least mean squares (LMS) technique to reduce noise in signals is well established [13], where an FIR filter is trained to reduce the noise in a signal [30] with the help of one or more reference signals. This has been shown to be effective against EOG by using as a reference for both the horizontal and vertical EOG to remove the artefacts from an EEG [15] but requires additional conventional electrodes placed above/below and left/right of the eyes. There have been various approaches to using neural networks to generate the signal (called here “remover”) which is used to eliminate the artefacts in the EEG signal [31]. While we use a standard encoder based deep net with a non-linear activation function, others used radial basis functions [32] or functional link neural networks (FLNN) to generate non-linear decision boundaries with non-linear functional expansion [33]. Even more computationally expensive is an approach where the shortcomings of the FLNN are reduced with the help of an adaptive, neural, fuzzy inference system [34]. In contrast, our deep network operates as a standard deep net and off-the-shelf optimised architectures are widely available. In particular, encoder structures are very popular across application domains and are readily available, for example, audio [35]. Thus, in terms of computational cost not only the standard encoder architecture is beneficial because of its wide availability but also makes it possible to directly use deep learning optimised hardware such as GPUs to perform the computations.

Traditionally, deep learning is a classifier and has been used to detect EEG artefacts with high accuracy of up to 90% [36–38] but not to remove the artefacts from the EEG. Deep learning can also assist ICA-based algorithms [6, 9] to identify the principal components which contain the EMG noise [39]. Direct removal of EMG noise has been investigated in the following network structures: fully connected neural networks, simple convolutional networks, complex convolutional networks, recurrent neural networks [40] and a new encoder/decoder-based architecture called DeepSeparator [41]. Only the fully connected network, the recurrent neural network and the DeepSeparator were stable during EMG removal. All these networks received the entire time series, outputted the entire time series, were trained offline and are thus not real-time. In contrast, our DNF performs continuous real-time training and filtering at the same time. These networks were not trained by an error between reference noise and the output of the filter but by an error between a clean EEG and the filter output [42, 43] which also served as the performance measure. Since clean EEGs are not readily available, they were, for example, generated with ICA from noisy EEGs [40]. The improvement of SNR before and after filtering was not stated by Zhang et al. [40] but the error between clean EEG and filter output settles at about 10%. Overall, as Urigüen et al. [44] noted, most EEG noise reduction studies are based only on synthetic signals and most only visually analyse their results.

With standard deep learning approaches [40, 41] where learning and filtering are done separately, there is always the risk of overfitting [45]. However, here learning is always “on” which means that the DNF is constantly adapting to new signals and noise contingencies. The learning rate of the DNF rather determines how quickly it adapts where a high learning rate could lead to “temporary overfitting” in particular on to large one-off artefacts whereas a low learning rate could not adapt to changing signal and noise contingencies.

The mechanical EEG electrode design is as old as the first EEG recordings [46] and the standard Ag/AgCl cup electrodes have been the main staple of EEG recordings ever since [47]. A major concern has always been the resistance between electrode and skin [48] which has an impact on the SNR of the EEG. The electrode resistance has become even more of a concern with the advent of BCI and consumer EEG headbands which favour dry electrodes [49]. Besides active electrodes [50] novel electrode designs promise to help reduce the electrode resistance [51, 52] in particular by using spring contact probes [53, 54]. However, these electrode designs only improve the SNR by a better skin/electrode contact but do not take into account the spatial distribution of signals versus noise which calls for compound electrodes.

The spatial distribution of electrodes has been in particular investigated with the rise of brain-computer interfaces (BCI) where often the user is actively using their muscles and thus creating a large amount of both EMG and movement artefacts [55]. It could be shown that the central average reference (CAR) and both small and large Laplacian montages [20] improve the SNR. This has been shown for Electrocardiogram (ECG) [18] by removing movement artefacts and for EEG [19]. Common to all approaches is the approximation and optimisation of a 2D spatial Laplace operator [17, 20, 56]. The more rings are employed at an optimal spacing the more efficient the operator will be. The calculation of the Laplacian is usually performed by the electrical summation of the EEG sources under each ring, digitisation and subtraction from each other. However, this assumes that every ring can perform a perfect analogue spatial averaging operation which is not the case in practice as electrode impedances will be inhomogeneous and changing over time. The analogue averaging over a ring can be overcome by measuring from a large number of electrodes from an EEG cap and then approximating the Laplace purely in software [16]—but this is computationally expensive and if using a standard EEG cap, it has its limitations in spatial resolution. On the other hand, the above discussed concentric ring electrode is the most feasible and practical hardware design [20], however, has the drawback of assuming perfect recording conditions that are only present in ideal biophysical models but not in real setups. To overcome the shortcomings of hardwired computations based on ideal models we use an adaptive algorithm to account for the imperfect nature of the electrodes and the dynamic changes of electrode resistance over time, in particular when using dry electrodes. By high-pass filtering the noise reference (i.e outer electrode) we can direct the learning algorithm towards the noise it should focus on which here was EMG noise.

A particular area of concern is the choice of adequate conductive electrode material. Bio-electrodes are in contact with the body and will, in turn, be exposed to biological electrolytes which can, over time, cause oxidation of the electrode and degrade the electrode’s quality [24, 57, 58]. It can be concluded that precious metals are the obvious choice for conductive material and many EEG electrodes utilise them to provide electrode conductivity [23, 59]. Due to the cost of such metals, a superficial, thin coating is usually applied to a cheaper backing material [52, 60], to provide high conductivity, good chemical stability and structural support for the electrode, simultaneously minimising the cost [61]. The conductive layer selected for the design discussed in this paper was also Ag/AgCl and was selected due to its high conductivity [52], chemical and electrical stability [24] and relative manufacturing simplicity as it can be printed as an ink [52, 62].

## Conclusion

To our knowledge, we are the first to perform simultaneous learning and noise reduction in real-time with a deep neural network without the classical sequential process of training first and then filtering. Specifically for removing EMG from EEG we have developed a novel electrode which in conjunction with the real-time deep learning algorithm implements a constantly adapting spatial Laplace filter. As a proof of concept, we have used data of 20 subjects performing a jaw-clench to produce easily identifiable EMG signals. Future research will focus on more realistic scenarios of EMG noise, for example playing a video game or performing a manual task where noise levels change dynamically which requires possibly an adaptive learning rate as used by variable step size LMS filters [63]. We will also investigate other symmetrical activation functions suitable for signal processing which are less computationally expensive, yield faster convergence and are robust against vanishing gradients. Generally, the DNF is also applicable to other domains such as noise cancelling headphones and will be addressed in the future.

## Supporting information

S1 Appendix DNF filtering with simulated EEG and EMG. (PDF)
