# Supplementary material for: Real-time noise cancellation with deep learning
Source: PLoS One. 2022 Nov 21;17(11):e0277974. doi: 10.1371/journal.pone.0277974 (PMC9678292; doi:10.1371/journal.pone.0277974)
Supplement: S1 Appendix — (PDF) [file pone.0277974.s001.pdf]

Real-time noise cancellation with Deep Learning

Bernd Porr, Sama Daryanavard, Lucía Muñoz Bohollo, Henry Cowan, Ravinder Dahiya

DNF filtering with simulated EEG and EMG

For the simulation, the pure EEG signal and the pure EMG noise are both generated artificially. For this, we use two uncorrelated random Gaussian noise sources for both EEG and EMG, filter them appropriately and then add them together. This has the advantage that the SNR can directly be calculated by cross-correlating the artificially generated pure EEG $c[n]$ with the output of the DNF $e[n]$ to obtain the pure EEG amplitude after DNF filtering without resorting to P300 for pure signal:

$$c_{\text{amplitude}} = \max_{m=-N \ldots N} \sqrt{\frac{1}{N} \left| \sum_{n=0}^{N-1} c[n-m] \cdot e[n] \right|} \tag{1}$$

where N is the number of samples of the recording and m an index which shifts the signals $e[n]$ and $c[n]$ against each other until they reach a maximum resulting in the amplitude $c_{\text{amplitude}}$ of the pure EEG at the output of the DNF. The simulation code uses the same DNF filter code [1] as for the real EEG data. Only the code segment that generates the simulated EEG, EMG, and the analysis using cross-correlation instead of P300 has been adapted but otherwise kept identical.

We first describe the pure EEG model: Data from Whitham et al. 2007 [2,3] of six EEG power spectra under complete paralysis (Fig. AA) has been used to create a model of pure EEG by filtering random Gaussian noise at a standard deviation of 40 μV with a 2nd order Butterworth low-pass filter with a cutoff frequency of $f_c = 17$ Hz ignoring DC (Fig. AA1) and alpha activity (Fig. AA2) because our subjects had their eyes open in contrast to the paralysed ones. To keep the model simple, it is assumed that the low-pass filtered Gaussian noise is the pure EEG signal $c[n]$ without any additional EEG-background activity.

The EMG signal model needs to reflect the spectrum of the jaw clench and is determined by taking the Fourier transform of 7 chunks of EMG containing jaw clenches from subject 10 (Fig. AC) which shows that below 20 Hz the power of the EMG rapidly declines [2]. When combining the jaw clenches, the low end at $f < 3$ Hz is ignored as it is not EMG but baseline shifts (Fig. AC1). Fig AB shows the generation of the simulated EMG: random Gaussian noise is generated at 15 μV, then boosted every 15 sec for one second by a factor of 5 to simulate the jaw clench and then filtered with a bandpass filter with centre frequency of $f_c = 50$ Hz and bandwidth of ± 35 Hz.

Fig. AB shows how the pure EEG $c[n]$ and EMG noise $r[n]$ are combined to arrive at the simulated signals for the inner electrode $d[n]$ and outer ring $x[n]$. To factor in the fact that EEG spills over from the inner electrode to the outer ring electrode, a cross-talk coefficient of $\alpha = 0.4$ has been selected, as introduced in Eq.2. As mentioned in the main part, this spillover of the EEG signal reduces the signal amplitude at the output of the DNF, as the DNF treats anything at the outer ring electrode $x[n]$ as noise. $\alpha = 0.4$ was chosen to arrive at a similar reduction of the filtered EEG amplitude during the simulation from 10 μV to 7.5 μV as observed for the real data (Fig. 3A, B). Remember that there is no need to measure P300 as the pure EEG is known here through simulation. The final simulated electrode signals are now calculated as:

$$d[n] = r[n] + c[n] \quad \text{Inner electrode: signal + noise} \tag{2}$$

$$x[n] = r[n] + \alpha \cdot c[n] \quad \text{Outer ring electrode: noise reference} \tag{3}$$

Fig. AD-I show the simulation results matching Fig. 2 and Fig. 4 in the main part of the paper. The removal of the simulated jaw clenches in Fig. AD,$e[n]$ follows clearly a similar development of

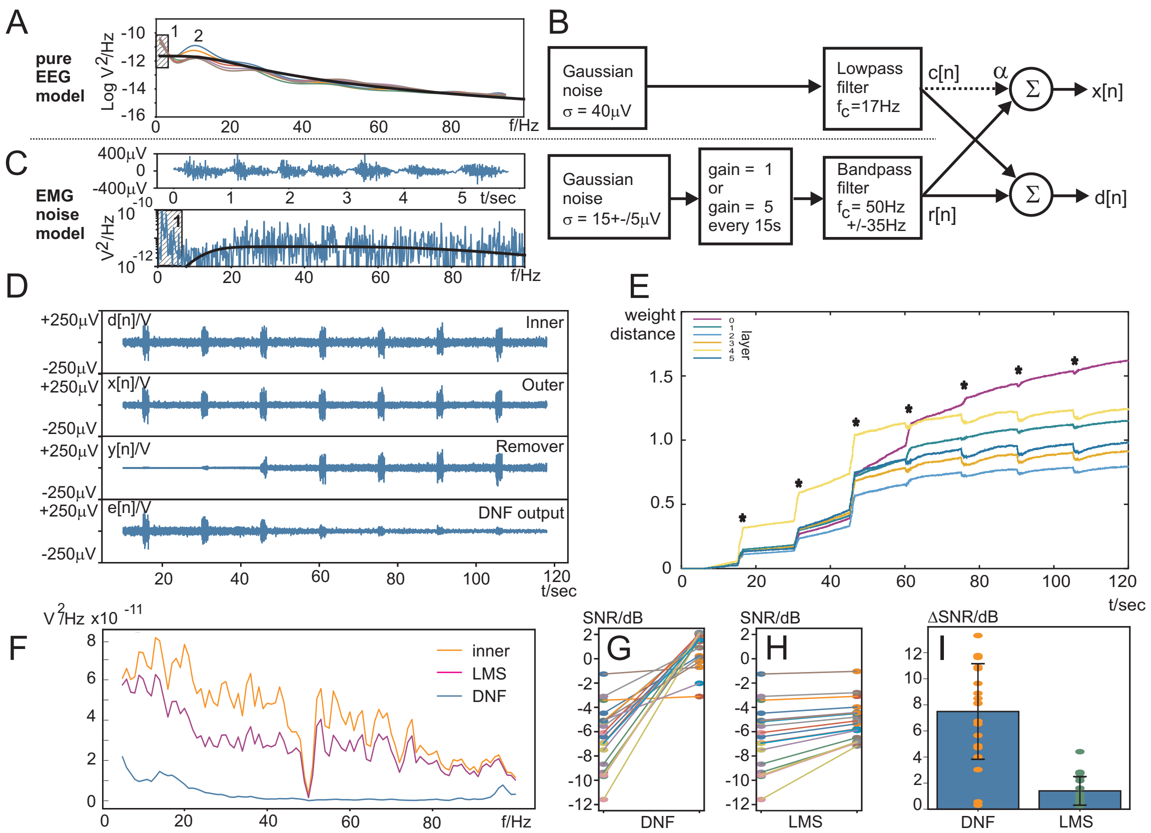

Fig A. Simulation results. A: Pure EEG data from Whitam et al. 2007 (thin, coloured lines) and 2nd order Butterworth approximation (thick, black line). B: Signal (EEG, $c[n]$) and noise (EMG, $r[n]$) model to generate the inner $d[n]$ and outer electrode $x[n]$ signals. $\alpha = 0.4$ simulates the spillover of the EEG signal into the outer noise reference electrode $x[n]$. C: Seven jaw clenches and its Fourier transform. The thick black line in the Fourier spectrum is the Butterworth bandpass approximation. D: Results of the simulation (matching Fig 2A in the main part of the paper) showing the inner electrode signal $d[n]$, the outer ring electrode $x[n]$, the remover $y[n]$ and the output of the DNF $e[n]$. E: weight development of the weights per layer matching Fig 2B. The "*" indicate the simulated jaw clenches. F: Noise power density in bins of 1 Hz at the inner electrode $d[n]$, at the output of the DNF $e[n]$ and at the output of the standard LMS-based adaptive FIR filter (matching Fig. 4A). G: SNR in dB at the inner electrode $d[n]$ and the output $e[n]$ of the DNF for 20 simulations. H: SNR in dB for the standard LMS-based adaptive FIR filter. I: The SNR differences from G) and H) for DNF and the LMS-based FIR filter.

the removal of the real jaw clenches in Fig 2A: the remover $y[n]$ grows in amplitude and then successfully removes the jaw clench at the output $e[n]$ of the DNF. The weight development of the simulation in Fig. AE follows also a similar development as the one with the real data in Fig 2B. Surprisingly, the real data makes the network converge faster than the simulation. The spectral distribution of the output of the DNF in Fig. AF is also smooth as in the real data in Fig. 4A and similarly, the LMS filter performs worse than the DNF. This is also confirmed quantitatively by the signal-to-noise analysis. The SNR analysis over 20 simulated subjects with different EMG noise amplitudes of $15\mu V \pm 5\mu V$ reveals a very similar performance between simulation and real data. As for the real data, the signals with the lowest SNR benefit most and those with already high SNR benefit less; this is as expected. Both our new DNF ($p = 0.000001$) and the LMS-tuned adaptive FIR filter ($p = 0.000023$) significantly improved the SNR but as for the real data the DNF is significantly better than the LMS filter ($p = 0.000001$). Overall, the simulation results confirm and validate those obtained with real data.

References

1. Porr B, Daryanavard S, Cowan H, Dahiya R. Deep Neuronal Filter: Real-time noise cancellation with Deep Learning; 2022. Available from: `https://doi.org/10.5281/zenodo.7100537`.

2. Whitham EM, Pope KJ, Fitzgibbon SP, Lewis T, Clark CR, Loveless S, et al. Scalp electrical recording during paralysis: quantitative evidence that EEG frequencies above 20 Hz are contaminated by EMG. Clinical neurophysiology : official journal of the International Federation of Clinical Neurophysiology. 2007;118(8):1877–1888. doi:10.1016/j.clinph.2007.04.027.

3. Porr B. Pure EEG power during paralysis; 2022. Available from: `https://doi.org/10.5281/zenodo.7038831`.
